# Cardiac remodelling and functional status after cardiac resynchronization therapy: comparison between de-novo implantation and upgrade from right ventricular pacing

**DOI:** 10.1093/eschf/xvag183

**Published:** 2026-06-26

**Authors:** Henrik Laurits Bjerre, Emil Anton Frandsen, Anders Sommer, Charlotte Stephansen, Niels Risum, Steen Hvitfeldt Poulsen, Jens Cosedis Nielsen, Mads Brix Kronborg

**Affiliations:** Department of Cardiology, Aarhus University Hospital, Palle Juul-Jensens Boulevard 99, 8200 Aarhus N, Denmark; Department of Clinical Medicine, Aarhus University, Palle Juul-Jensens Boulevard 82, 8200 Aarhus N, Denmark; Department of Cardiology, Copenhagen University Hospital—Rigshospitalet, Blegdamsvej 9, 2100 København, Denmark; Department of Cardiology, Aarhus University Hospital, Palle Juul-Jensens Boulevard 99, 8200 Aarhus N, Denmark; Department of Cardiology, Aalborg University Hospital, Hobrovej 18-22, 9000 Aalborg, Denmark; Department of Cardiology, Copenhagen University Hospital—Rigshospitalet, Blegdamsvej 9, 2100 København, Denmark; Department of Cardiology, Aarhus University Hospital, Palle Juul-Jensens Boulevard 99, 8200 Aarhus N, Denmark; Department of Clinical Medicine, Aarhus University, Palle Juul-Jensens Boulevard 82, 8200 Aarhus N, Denmark; Department of Cardiology, Aarhus University Hospital, Palle Juul-Jensens Boulevard 99, 8200 Aarhus N, Denmark; Department of Clinical Medicine, Aarhus University, Palle Juul-Jensens Boulevard 82, 8200 Aarhus N, Denmark; Department of Cardiology, Aarhus University Hospital, Palle Juul-Jensens Boulevard 99, 8200 Aarhus N, Denmark; Department of Clinical Medicine, Aarhus University, Palle Juul-Jensens Boulevard 82, 8200 Aarhus N, Denmark

**Keywords:** Cardiac device, Cardiac resynchronization therapy, CRT, Biventricular pacing, RV pacing, Upgrade

## Abstract

**Introduction:**

Patients with heart failure induced by chronic right ventricular (RV) pacing commonly undergo upgrade to cardiac resynchronization therapy (CRT). We aimed to compare the effect size of improvement in cardiac function and functional capacity between patients with RV pacing and intrinsic conduction at baseline.

**Methods:**

We included patients from the ImagingCRT, ElectroCRT and DANISH-CRT trials aged ≥40 years with left ventricular ejection fraction (LVEF) ≤35% and prolonged QRS. The primary endpoint was change in left ventricular end-systolic volume (LVESV) from baseline to 6-month follow-up. Secondary endpoints included change in QRS duration, echocardiographic measures (LVEF, left ventricular end-diastolic volume, left ventricular mass index, and left atrial volume index), N-terminal prohormone of brain natriuretic peptide, six-minute walk test, quality of life, New York Heart Association functional class and loop diuretic use.

**Results:**

We included 592 patients [mean age 70.7 ± 9 years; 24.8% women; mean LVEF 27 ± 6%; 97 (16.4%) with RV pacing and 495 (83.6%) with intrinsic conduction at baseline]. We found significant relative reduction in LVESV in patients with RV pacing (−34 ± 25%) and intrinsic conduction (−32 ± 26%) with no difference between the groups [mean difference −2%, 95% CI (−8; 4), *P* = .469]. Absolute LVEF improvement was 14 ± 9% and 13 ± 10%, respectively. Reduction in QRS duration was larger with RV pacing compared with intrinsic conduction (−38 ± 26 vs −26 ± 24 ms). We found significant improvements in the remaining secondary endpoints, with no difference between the groups.

**Conclusion:**

Patients with RV paced QRS morphology who are upgraded to CRT derive substantial improvement in cardiac function and functional capacity, comparable to patients with intrinsic conduction undergoing CRT.

## Graphical Abstract

**Graphical Abstract:**
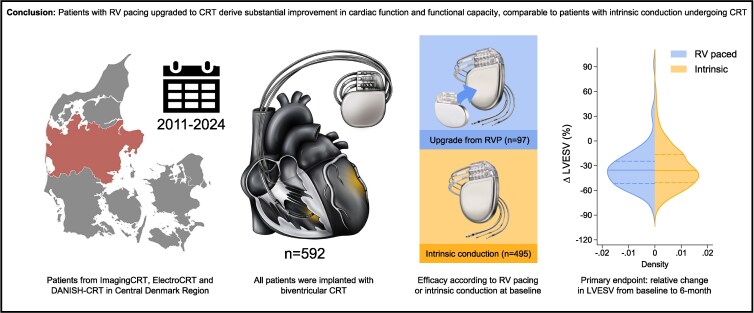

Effect of biventricular cardiac resynchronization therapy (CRT) in patients with intrinsic conduction and right ventricular (RV) pacing at baseline. Patients with RV pacing at baseline who were upgraded to CRT derived substantial improvement in cardiac remodelling and functional capacity from baseline to 6 months follow-up. The effect size was comparable to patients with intrinsic conduction at baseline undergoing CRT.

## Introduction

Chronic right ventricular (RV) pacing can lead to worsening of left ventricular (LV) function and induce symptomatic heart failure (HF).^1–3^ Patients with pacing-induced HF may be upgraded to a biventricular cardiac resynchronization therapy (CRT) device by adding an epicardial LV lead in a coronary sinus tributary vein and replacing the generator.^4^ Most evidence on the beneficial effects from CRT originates from patients with intrinsic conduction undergoing *de novo* implantation, while less research has investigated efficacy of CRT in patients who are upgraded from a previous pacemaker or implantable cardioverter defibrillator (ICD) due to HF considered induced by RV pacing.^5^ A recent meta-analysis^6^ with six (*n* = 161) small randomized controlled trials (RCT) and 47 observational studies (*n* = 2,644) found improved LVEF and functional capacity following upgrade from a pacemaker or ICD to a biventricular CRT device. Likewise, the BUDAPEST-CRT^7^ trial showed that upgrade to a CRT defibrillator (CRT-D) was superior to an ICD on the primary composite endpoint of all-cause mortality, HF hospitalization and cardiac remodelling. Although this demonstrates a beneficial effect of upgrade procedures, studies have reported conflicting results on whether the size of this effect is comparable to that seen in *de novo* procedures.^8–12^ The interpretation of these results is made additionally complex by several advancements over the past 25 years, including different patient selection and technological improvements such as quadripolar leads, optimization algorithms and targeting strategies for lead positioning, as well as evolution in pharmacological HF treatment. In a contemporary setting, it therefore remains unclear whether patients with an RV paced QRS who are upgraded to CRT derive similar short-term benefits as patients with intrinsic conduction who undergo a *de novo* CRT procedure. In a large contemporary CRT cohort, this study aimed to evaluate the effect size of six-month benefits in terms of cardiac remodelling and functional capacity in patients with RV paced QRS and intrinsic conduction (*Graphical abstract*).

## Methods

### Design and study population

This region-wide prospective cohort study comprised patients enrolled in three RCTs; ‘Multimodality Imaging-guided Left Ventricular Lead Placement in Cardiac Resynchronization Therapy’ (ImagingCRT),^13^ ‘Electrically vs Imaging-guided Implant of the LV Lead in Cardiac Resynchronization Therapy’ (ElectroCRT)^14^ and ‘Does Electric Targeted LV Lead Positioning Improve Outcome in Patients With Heart Failure and Prolonged QRS’ (DANISH-CRT).^15^ All trials were approved by The Central Denmark Regional Committee on Health Research Ethics (files no. 20100245, 1-10-72-230-14 and 1-10-72-330-16, respectively) and the Danish Data Protection Agency (files no. 2011-41-6017, 1-16-02-622-14 and 1-16-02-660-16, respectively) and conformed to the declaration of Helsinki. All patients provided written informed consent. Patients were referred from one of five hospitals in The Central Denmark Region, comprising a population of 1.3 million, and all implantations were performed at Aarhus University Hospital, Denmark. Patients were enrolled in the respective trials from 2011–2014, 2015–2017 and 2018–2024. The inclusion criteria have been previously described.^15–17^ Briefly, the trials enrolled patients aged ≥40 years with symptomatic HF corresponding to New York Heart Association (NYHA) functional class II-IVa with LVEF ≤35% on echocardiography despite optimal medical therapy. Eligibility with respect to QRS duration varied slightly between trials: left bundle branch block (LBBB) with QRS ≥120 ms or RV paced QRS ≥180 ms in ImagingCRT and ElectroCRT, and true LBBB with QRS ≥130 ms, LBBB-like, right bundle branch block (RBBB) or interventricular conduction delay (IVCD) all with QRS≥150 ms or RV paced QRS with >50% RV pacing in the DANISH-CRT trial. Trial participants were excluded from this pooled cohort study if echocardiograms were not available at baseline and follow-up. Patients were followed from baseline to 6 months follow-up. The LV lead was guided to the site of latest mechanical activation, latest electrical activation or according to the operator’s preference depending on the assigned randomization arm in each respective trial. The RV lead was aimed towards a mid-septal position, with post-implant optimization of AV- and VV-delays according to trial-specific protocols using echocardiographic and/or electrocardiographic guidance. Optimization protocols were identical for patients with RV pacing and intrinsic conduction at baseline.^15–17^ The highest achievable degree of biventricular pacing was prioritized rather than fusion with intrinsic conduction (i.e. LV pacing only or triple fusion).

### Endpoints

The primary endpoint was relative change in LV end-systolic volume (LVESV) from baseline to 6-month follow-up. Secondary endpoints included change in LV end-diastolic volumes (LVEDV), LVEF, left atrial volume index (LAVI), LV mass index (LVMI), QRS duration on surface ECG, distance on six-minute walk test (6MWT), N-terminal prohormone of brain natriuretic peptide (NT-proBNP), NYHA functional class, quality of life (QoL) and use of loop diuretics. We also evaluated conventional measures of CRT response defined as ≥15% relative reduction in LVESV, ≥5% absolute increase in LVEF or ≥1 reduction in NYHA functional class. Echocardiograms were analysed in core-lab/single-centre setup and blinded for other echocardiograms from the same patient. All echocardiograms were performed on General Electric (GE) healthcare scanners (Vivid E9 in ImagingCRT and ElectroCRT and Vivid E95 in DANISH-CRT) and analysed using identical software (EchoPac, GE healthcare). LVESV, LVEDV, and LVEF were assessed using the Simpson’s biplane method. LAVI was measured using the method of discs, and LVMI was measured using the American Society of Echocardiography modified equation, both indexed to the body surface area (Du Bois’ formula). LAVI and LVMI measurements were only available for patients enrolled in DANISH-CRT while NT-proBNP at follow-up and 6MWT at baseline only were measured in ImagingCRT and ElectroCRT. QoL was assessed with the 21-item Minnesota Living with Heart Failure questionnaire^18^ (range 0–105, lower values indicate better QoL) in ImagingCRT and ElectroCRT and with the 12-item Kansas City Cardiomyopathy Questionnaire^19^ (range 0–100, higher values indicate better QoL) in DANISH-CRT. To enable comparison across the instruments, we used the standardized ΔZ-score relative to the baseline standard deviation (SD) with positive values reflecting improvement irrespective of questionnaires.

### Statistics

All demographic, procedural and medical data were collected and stored in electronic web-based case report forms (eCRF). Continuous variables were reported as means and standard deviations (SD) in case of normal distribution and as medians and interquartile range (IQR) in absence of normal distribution. Normality was assessed by visual inspection of histograms and Q-Q plots. Categorical variables were presented as absolute numbers and percentages. Endpoints were compared using unpaired *t*-test for continuous variables in case of normal distribution, Wilcoxon rank-sum test in absence of normal distribution and Fisher’s Exact test for categorical variables. Endpoints were presented as mean difference with 95% confidence intervals (CI) or as median ratios after back-transformation from log-transformation. The primary endpoint was evaluated using a multivariable linear regression model to explore the influence of measured potential confounders. Covariates were selected *a priori* using directed acyclic graphs (DAG). Potential effect-modification by trial was explored by testing the interaction between trial and baseline QRS morphology (RV pacing vs intrinsic conduction). We used box plots with medians, IQR and upper and lower adjacent values to visualize distribution of continuous endpoint variables. For the reported *P*-values in the box plots we used Wilcoxon rank-sum test for between-group differences and Wilcoxon signed rank test for paired analysis of changes from baseline to 6-month follow-up. We performed sensitivity analysis for all endpoints with exclusion of (i) patients with intrinsic non-LBBB QRS morphology and (ii) patients LVEF >35% at baseline. For the comparison of RV pacing, intrinsic LBBB and intrinsic non-LBBB we used one-way ANOVA and subsequent pairwise comparison with Bonferroni correction, or Kruskal–Wallis test pending normality. Lastly, we conducted hypothesis-generating subgroup analyses according to pre-existing device type (pacemaker or ICD) and duration of RV pacing (earlier or later than the median duration from the patient’s first device to CRT upgrade). Statistical analysis was performed using Stata (version 18; StataCorp).

## Results

We identified 727 patients enrolled from April 2011 to June 2024. We excluded 135 patients who did not have available echocardiograms from both baseline and 6-month follow-up. Of the total 592 patients included, 97 (16.4%) were upgraded from a pre-existing pacemaker or ICD and had RV paced QRS complex at baseline. The remaining 495 (83.6%) were *de novo* recipients or patients who were upgraded from a pre-existing device but had intrinsic conduction at baseline. In patients with intrinsic conduction, 476 (96%) had LBBB QRS morphology while only 19 (4%) had LBBB-like morphology, right bundle branch block (RBBB) or IVCD. Patients with RV paced QRS at baseline were upgraded from a pacemaker (81%) more often than an ICD (19%). In contrast, those with a pre-existing device, but who had intrinsic conduction, were upgraded from an ICD (78%) more often than a pacemaker (22%). Patients with RV paced QRS were older and more likely to have ischaemic heart disease and atrial fibrillation compared with patients with intrinsic conduction (*Table 1*). Both patient groups had substantial use of guideline-directed medical therapy (GDMT) with prescription rates of 85–88% for betablockers and 94–96% for angiotensin converting enzyme inhibitors (ACEi), angiotensin-II-receptor blockers (ARB) or neprilysin inhibitors (ARNi), with 27–36% of patients achieving target dose. However, more patients with intrinsic conduction than with RV pacing were treated with spironolactone or eplerenone (*Table 2*). At baseline, patients with RV paced QRS had longer QRS duration but better cardiac function (lower LVESV, lower LVEDV and slightly higher LVEF) compared with patients with intrinsic conduction (*Table 3*). They also had lower functional capacity with higher NYHA functional class and shorter distance on the 6MWT. Patients with RV paced QRS had greater reduction in QRS duration than patients with intrinsic conduction (*Table 3*).

**Table 1:** Baseline characteristics

	Total (*n* = 592)	RV paced (*n* = 97)	Intrinsic (*n* = 495)	*P*-value
Age, years	73 (64–78)	75 (71–80)	72 (63–78)	<.001
Sex, male	445 (75)	79 (81)	366 (74)	.118
BMI, kg/cm^2^	27 (5)	28 (5)	27 (5)	.405
Ischaemic heart disease	271 (46)	55 (57)	216 (44)	.017
Hypertension	304 (52)	58 (61)	246 (50)	.055
Diabetes	164 (28)	25 (26)	139 (28)	.642
Chronic obstructive pulmonary disease	57 (10)	6 (6)	51 (10)	.205
Atrial fibrillation				<.001
Persistent or permanent	92 (16)	35 (36)	57 (12)	
Paroxysmal	119 (20)	25 (26)	94 (19)	
Previous device	161 (27)	97 (100)	64 (13)	<.001
Pacemaker	93 (58)	79 (81)	14 (22)	<.001
Original indication				
AV block	48 (52)	43 (54)	5 (36)	
Sinus node dysfunction	26 (28)	19 (24)	7 (50)	
Permanent atrial fibrillation	11 (12)	11 (14)	0 (0)	
Other/unknown	8 (9)	6 (6)	2 (14)	
ICD	68 (42)	18 (19)	50 (78)	<.001
Original indication				
Primary prevention	38 (56)	10 (56)	28 (56)	
Secondary prevention	30 (44)	8 (44)	22 (44)	
RV-pace% on last interrogation, %	92 (1–99)	99 (90–100)	1 (0–8)	
Time since previous device, years	4.0 (1.8–6.7)	3.8 (1.7–6.7)	4.5 (2.5–6.8)	.167
Time since first device, years	5.0 (2.3–8.9)	4.5 (1.9–7.6)	5.7 (3.1–9.4)	.071
Systolic blood pressure, mmHg	122 (110–135)	123 (113–137)	122 (110–134)	.218
Diastolic blood pressure, mmHg	71 (65–79)	71 (64–80)	71 (65–78)	.440
Creatinine	95 (80–121)	98 (84–120)	95 (79–122)	.085
eGFR				.155
<29	27 (5)	8 (8)	19 (4)	
30–60	237 (40)	38 (40)	199 (40)	
>60	325 (55)	50 (52)	275 (56)	
Haemoglobin	8 (8–9)	8 (8–9)	8 (8–9)	.892
Procedure time, min	90 (68–104)	90 (64–105)	90 (68–103)	.694
X-ray time, min	21 (11–25)	21 (11–25)	21 (11–25)	.617

Data are presented median (IQR) for continuous variables and *n* (%) for categorical measures. Between-group differences are assessed using the Wilcoxon rank-sum test for continuous variables and Pearson’s chi-square test for categorical variables. BBB, bundle branch block; BMI, body mass index; eGFR, estimated glomerular filtration rate; IQR, interquartile range; RV, right ventricular.

**Table 2:** Medication at baseline

	Total (*n* = 592)	RV paced (*n* = 97)	Intrinsic (*n* = 495)	*P*-value
Beta blocker	524 (89)	82 (85)	442 (89)	.221
<50% of TD	191 (32)	35 (36)	156 (32)	
50–99% of TD	157 (27)	21 (22)	136 (27)	
≥100% of TD	176 (30)	26 (27)	150 (30)	.317
ACEi, ARB or ARNi	555 (94)	93 (96)	462 (93)	.491
<50% of TD	194 (33)	37 (38)	157 (32)	
50–99% of TD	151 (26)	26 (27)	125 (25)	
≥100% of TD	208 (35)	30 (31)	178 (36)	.504
Spironolactone/eplerenone	346 (59)	44 (45)	302 (61)	.005
<50% of TD	39 (7)	6 (6)	33 (7)	
50–99% of TD	268 (45)	32 (33)	236 (48)	
≥100% of TD	39 (7)	6 (6)	33 (7)	.031
SGLT2 inhibitor	88 (15)	14 (15)	74 (15)	1.000
Loop diuretic	409 (69)	68 (71)	341 (69)	.809
Furosemide equipotent dose	60 (40–120)	60 (40–120)	60 (40–120)	.922
Thiazide diuretic	23 (4)	5 (5)	18 (4)	.562
Calcium channel blocker	43 (7)	14 (15)	29 (6)	.008
Digoxin	32 (5)	5 (5)	27 (5)	1.000
Amiodarone	65 (11)	10 (10)	55 (11)	1.000
Antiplatelet drug	318 (54)	43 (44)	275 (56)	.045
Oral anticoagulation	207 (35)	52 (54)	155 (31)	<.001

Categorical data are presented as *n* (%) and between-group differences were assessed using Fisher’s exact test. Continuous variable is presented as median (IQR) and between-group difference was assessed using Wilcoxon rank-sum test. ACEi, angiotensin converting enzyme inhibitor; ARB, angiotensin-II-receptor blocker, SGLT2, sodium-glucose-linked-transporter-2; TD, target dose.

**Table 3:** Endpoints at baseline and follow-up and change from baseline to follow-up for patients with RV pacing and intrinsic conduction and between-group difference with 95% confidence intervals

	RV paced (*n* = 97)	Intrinsic (*n* = 495)	Difference (95% CI)	*P*-value
Biventricular pacing, %				
Follow-up	99 (97–99)	99 (96–99)	—	.092
QRS duration, ms				
Baseline	185 ± 21	166 ± 18	18 (15; 23)	<.001
Follow-up	146 ± 22	140 ± 20	6 (2; 11)	.006
Absolute change	−38 ± 26	−26 ± 24	−12 (−17; −6)	<.001
LVESV, mL				
Baseline	131 ± 52	157 ± 69	−26 (−40; −11)	.001
Follow-up	82 ± 37	104 ± 58	−21 (−33; −9)	.001
Absolute change	−48 ± 43	−51 ± 51	5 (−6; 15)	.402
Relative change (%)	−34 ± 25	−32 ± 26	−2 (−8; 4)	.469
LVEDV, mL				
Baseline	182 ± 63	211 ± 82	−29 (−47; −12)	.001
Follow-up	141 ± 50	165 ± 70	−24 (−39; −10)	.001
Absolute change	−41 ± 50	−46 ± 60	5 (−7; 18)	.400
Relative change (%)	−20 ± 23	−19 ± 25	−1 (−6; 5)	.797
LVEF, %				
Baseline	29 ± 6	27 ± 6	2 (1; 3)	.004
Follow-up	43 ± 9	40 ± 10	3 (1; 5)	.007
Absolute change	14 ± 9	13 ± 10	1 (−1; 3)	.356
LV mass index^a^, g/m^2^				
Baseline	121 ± 25	126 ± 34	−5 (−15; 5)	.322
Follow-up	108 ± 26	111 ± 34	−3 (−13; 7)	.525
Absolute change	−13 ± 25	−14 ± 31	2 (−7; 10)	.697
LA volume index^a^, mL/m^2^				
Baseline	42 ± 19	37 ± 17	5 (0; 11)	.069
Follow-up	43 ± 21	36 ± 16	7 (2; 13)	.010
Absolute change	1 ± 12	−1 ± 11	2 (−1; 6)	.195
NT-proBNP^b,c^, ng/L				
Baseline	1773 (984–3049)	1319 (637–3115)	1.29 (0.99; 1.67)	.053
Follow-up	1163 (786–1901)	687 (283–1602)	1.74 (1.10; 2.75)	.019
Relative change (GMR)	0.56 (0.43–0.72)	0. 48 (0.42–0.54)	1.17 (0.84; 1.64)	.353
6MWT, m				
Baseline^b^	342 ± 143	395 ± 111	−53 (−90; −11)	.014
Follow-up	401 ± 100	438 ± 100	−37 (−62; −13)	.003
Absolute change	58 ± 65	41 ± 65	17 (−7; 42)	.160
NYHA functional class				
Baseline I/II/III or IVa, n (%)	0(0)/42(43)/55(57)	0(0)/255(52)/240(48)	—	.299
Follow-up I/II/III or IVa, n (%)	25(26)/53(56)/17(18)	165(33)/270(55)/59(12)	—	.297
≥1 improvement, n (%)	57 (60)	320 (65)	—	.414
Quality of Life				
*MLWHF* ^b^				
Baseline	36 ± 21	34 ± 22	2 (−5; 10)	.536
Follow-up	21 ± 19	20 ± 19	1 (−5; 8)	.706
Absolute change	−16 ± 25	−14 ± 18	−2 (−8; 5)	.553
*KCCQ12* ^a^				
Baseline	54 ± 24	57 ± 19	−4 (−10; 2)	.227
Follow-up	68 ± 20	72 ± 19	−4 (−10; 1)	.138
Absolute change	15 ± 22	14 ± 18	1 (−4; 7)	.645
*Standardized change, Δ z-score*	0.74 ± 1.12	0.65 ± 0.86	0.09 (−0.11; 0.30)	.387
Loop diuretics, mg				
Baseline	60 (40–120)	60 (40–80)	—	
Follow-up	40 (40–100)	40 (40–80)	—	
Dose reduction, *n* (%)	23 (33)	102 (29)	—	.473

Data are presented as mean (SD) or median (IQR) for continuous variables pending normal distribution and *n* (%) for categorical measures. Between-group differences are assessed using unpaired *t*-test in case of normal distribution, the Wilcoxon rank-sum test in absence of normal distribution and Pearson’s chi-square test for categorical variables. Within-group differences are assessed using the paired *t*-test in case of normal distribution and the Wilcoxon signed rank test in absence of normal distribution.

^a^Data only available from the DANISH-CRT trial (*n* = 300).

^b^Data only available from the Imaging CRT and Electro CRT trials (*n* = 292).

^c^The median ratio was calculated as the geometrical mean ratio = the mean difference on the log scale. The median ratio may differ from the ratio of raw medians due to distributional asymmetry. QoL was assessed with MLWHF (range 0–105, lower scores reflect better QoL) and KCCQ12 (range 0–100, higher values indicate better QoL) and standardized Δ *z*-score using the baseline standard deviation (positive values indicate improvement) was used to enable comparison across instruments. 6MWT, Six-minute walk test; KCCQ12, Kansas City Cardiomyopathy Questionaire 12; LVEDV, left ventricular end-diastolic volume; LVEF, left ventricular ejection fraction; LVESV, left ventricular end-systolic volume; MLWHF, Minnesota Living with Heart Failure; NT-proBNP, N-terminal pro-Brain Natriuretic Peptide; NYHA, New York Heart Association.

**Figure 1:**
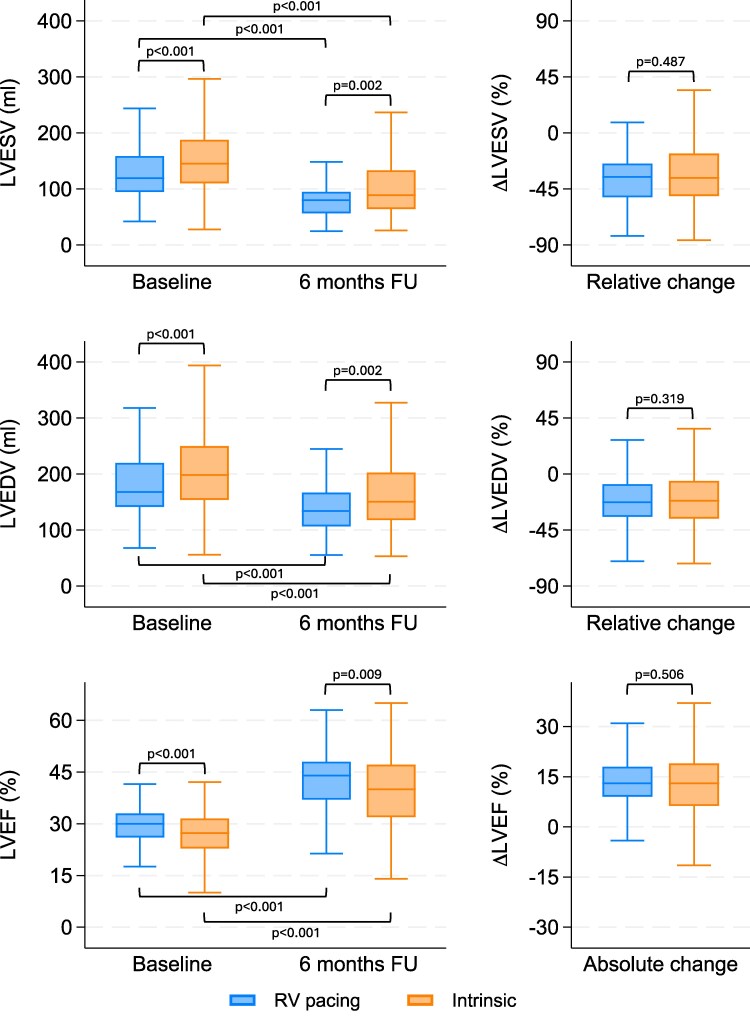
Boxplots showing median and IQR with upper and lower adjacent values representing the maximum value within 1.5 times the IQR at baseline and 6-month follow-up (left panel) and change from baseline to follow-up (right panel) for LVESV (upper panel), LVEDV, (middle panel) and LVEF (lower panel) for patients with RV pacing and intrinsic conduction. Values outside the upper and lower adjacent values are omitted due to data sensitivity (*n* < 5). Between-group differences were tested using the Wilcoxon rank-sum test and paired change from baseline to follow-up was tested using the Wilcoxon signed-rank test. IQR, interquartile range; LVEDV, left ventricular end-diastolic volume; LVEF, left ventricular ejection fraction; LVESV, left ventricular end-systolic volume

**Figure 2:**
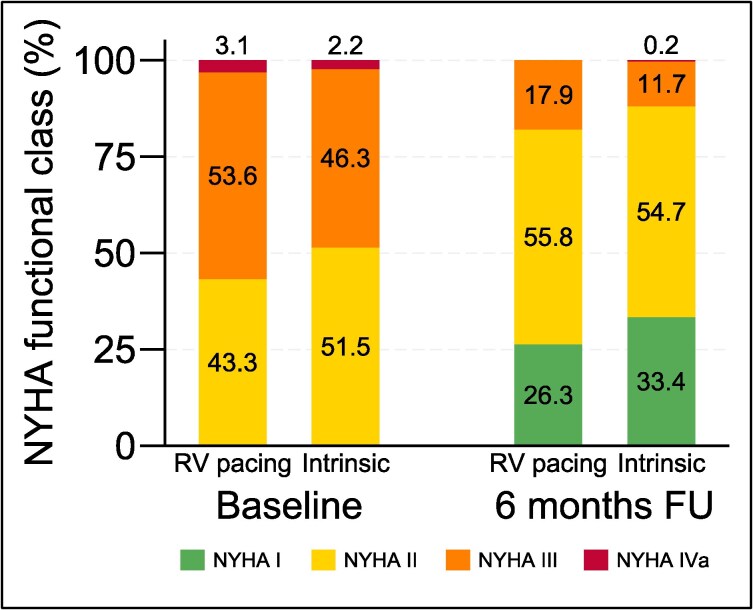
Distribution of NYHA functional class at baseline and follow-up for patients with RV pacing and intrinsic conduction. NYHA, New York Heart Association, RV, right ventricular

**Figure 3:**
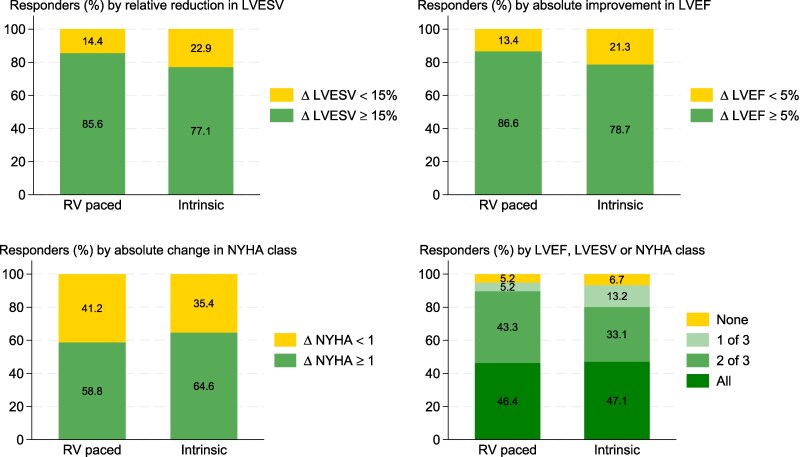
Percentage of patients classified as responders according to conventional definitions of CRT response including relative reduction in LVESV ≥15% (upper left), absolute increase in LVEF ≥5% (upper right), ≥1 improvement in NYHA functional class (lower left) or any of the previous parameters (lower right)

Regarding the primary endpoint (*Figure 1*, *Table 3*), we found a significant relative reduction in LVESV from baseline to 6-month follow-up for patients with RV pacing and intrinsic conduction (−34 ± 25% and −32 ± 26%, respectively) with no significant between-group difference [mean difference −2%, 95% CI (−8; 4), *P* = .469]. This was consistent in the multivariable linear regression model adjusting for age, sex, ischaemic heart disease, atrial fibrillation, QRS duration at baseline and trial [mean difference −2%, 95% CI (−8; 5), *P* = .633, Supplementary Table S1]. There was no evidence of effect-modification by trial (*P* = .65). Likewise, we found a significant reduction in LVEDV, LVMI, NT-proBNP and use of loop diuretics, and increase in LVEF and 6MWT distance, with no significant between-group differences. LAVI did not change from baseline to 6-month follow-up in any groups (*Table 3*). QoL improved significantly (mean standardized ΔZ-score 0.74 ± 1.11 and 0.65 ± 0.86 times the SD at baseline for RV pacing and intrinsic conduction, respectively) with no significant difference between the groups (*Table 3*). We found no differences between the groups in improvement on NYHA functional class (*Figure 2*). In terms of conventional definitions of CRT response, we observed slightly higher rates of echocardiographic response (84.5 vs 76.9%, *P* = .078 and 86.6 vs 78.7%, *P* = .095, for relative reduction in LVESV ≥15% and absolute improvement in LVEF ≥5%, respectively) and slightly lower response in ≥1 improvement in NYHA functional class (58.8% vs 64.6%, *P* = .299) with RV pacing compared with intrinsic conduction but none reached statistical significance (*Figure 3*).

Patients with non-LBBB intrinsic conduction had significantly less improvement from baseline to 6-month follow-up compared with both intrinsic LBBB and RV paced QRS on the primary (ΔLVESV −13 ± 24% vs −34 ± 25 vs −33 ± 26, respectively, *P* < .001) and secondary endpoints (Supplementary Table S2). Sensitivity analysis was consistent with the primary analysis on all endpoints when excluding patients with non-LBBB QRS morphology (*n* = 19, Supplementary Table S2) and patients with LVEF >35% at baseline (*n* = 30, Supplementary Table S3), respectively. Subgroup analyses suggested that patients with a pre-existing pacemaker experienced more extensive cardiac remodelling than patients with a previous ICD, with no differences in functional capacity (Supplementary Table S4). We found no differences in cardiac remodelling or functional capacity pending the duration of RV pacing (Supplementary Table S5).

## Discussion

In this prospective cohort study, we found that the 6-month effect size following CRT implantation in terms of improved cardiac function and functional capacity was similar for patients with RV pacing and patients with intrinsic conduction at baseline. These results were robust in sensitivity analyses where only patients with LBBB and with LVEF ≤35% were included. We also found that patients with LBBB-like, RBBB and IVCD seem to benefit less compared with patients with true LBBB and RV pacing even though they had significant QRS prolongation ≥150 ms. These findings are concordant with those of Wokhlu *et al*.^20^ (ΔLVEF was 7.7 ± 10.7 for RV pacing, 7.9 ± 10.2 for LBBB, 3.1 ± 8.5 for RBBB and 2.7 ± 7.3% for IVCD) although the effect size in our current study is larger. This difference likely reflects different patient selection, improved HF medication and CRT procedural development over the past 15 years. This includes lead types and positioning, device optimization algorithms and AV and VV delay optimization, which has been widely adopted by operators despite lack of decisive evidence from large trials. For example, AV and VV delay optimization was used in 50–60% of cases according to the CRT Survey II.^5^ Rickard *et al*.^12^ also found similar echocardiographic response following CRT between RV pacing and LBBB at baseline (ΔLVEF 9.6 ± 10.6 vs. 11.6 ± 12.2%) and Khursid *et al*.^11^ found an effect size in LVEF improvement similar to ours for patients with pacing-induced HF (LVEF increased from 29.3% to 45.3%). In contrast Vamos *et al*.^9^ reported a smaller improvement in cardiac function with RV pacing compared with LBBB (ΔLVEF 2.9 ± 9.0 vs 6.7 ± 9.4%, ΔLVEDV 0.0 ± 12.2 vs −3.5 ± 6.7 mL). The authors pointed to several possible explanations for this difference, including upgrade procedures being performed too late. However, in our subgroup analysis we found no difference in cardiac remodelling or functional capacity in patients with shorter compared with longer duration between the first devices to CRT upgrade. The authors also invoked higher procedural risk for upgrade procedures and that the ventricular activation pattern induced by RV pacing is inherently different than intrinsic LBBB. Although RV pacing resembles LBBB on surface ECG, studies have demonstrated differences between intrinsic LBBB and RV paced QRS morphology, including wider QRS complex, longer transseptal activation time, later activation of the LV free wall and changes in the site of the latest electrical activation.^21–24^ The LV activation pattern may also differ depending on the RV lead position however, the RV lead position does not seem to influence the risk of developing pacing-induced HF^25,26^ but determining the exact lead position solely based on fluoroscopy is notoriously difficult.^27^ Although RV pacing induces variable LV activation patterns which are distinctive from intrinsic LBBB, we found similar improvements in both conventional echocardiographic parameters and functional capacity. The majority of patients with RV pacing at baseline were upgraded from a pre-existing pacemaker rather than an ICD and patients upgraded from a pacemaker showed more extensive remodelling than those upgraded from an ICD. This likely reflects that in pacemaker recipients, who typically have preserved LVEF at the time of implantation and in whom dyssynchrony induced by RV pacing is the key element eliciting HF, LV function can be expected to recover substantially. In contrast, in patients that had an ICD implanted in already established HF with reduced LVEF, and subsequently have developed conduction disorder, RV pacing can be an innocent bystander, and reverse remodelling can be absent or limited to the level seen prior to development of the conduction disorder. To investigate this question, however, requires serial evaluations of LV functions over time, which was not available, and thus constitutes a limitation to this study. We also considered endpoint measurements which have not previously been reported in this setting, with similar reductions in both LVMI, NT-proBNP and use of loop diuretics. Interestingly we found no reduction in LAVI from baseline to 6-month follow-up, consistent with a previous study that utilized cardiac computed tomography for the measurement of atrial volumes and found a very small reduction in LAVI and no association between LAVI reduction and CRT response.^28^

Patients with RV pacing were older and had a higher burden of comorbidities at the time of CRT implantation and there was a tendency towards slightly better echocardiographic response and slightly worse improvement in NYHA functional class, although not statistically significant. This likely reflects that the comorbidities in patients with RV pacing limit their functional capacity despite prominent improvement in cardiac function. This consideration is supported by the higher NYHA functional class and shorter distance on 6MWT at follow-up. Importantly, NYHA functional has poor correlation with cardiac function and long-term outcomes and it is unknown if the similar short-term effect of CRT translates to similar long-term survival and freedom from HF hospitalization, as previous studies have reported both better,^10^ similar^29^ and worse^12,30^ long-term survival in patients undergoing upgrade compared with *de novo* procedures. In terms of direct clinical implications, our findings suggest that patients with RV paced QRS who undergo CRT upgrade can be informed to expect a benefit comparable to those receiving *de novo* CRT. This is important given that upgrade procedures are often considered more complex procedures, with higher risk of procedural risks and lead-related complications.^31–34^ For example, the rate of any complication in the BUDAPEST-CRT trial upgrade group was 25/211 (12.3%). The magnitude of this added procedural risk is uncertain^6^ and may be mitigated by procedural strategies.^35^ Ultimately, the clinical decision to upgrade a patient from RV pacing to CRT must balance the anticipated physiological improvement against the additional procedural risk, a question that should ideally be tested in a parallel design RCT.

Lastly, recent years have seen the introduction and widespread clinical adaptation of conduction system pacing (CSP), that utilizes His-Bundle pacing (HBP) or Left bundle branch area pacing (LBBAP) to pace the innate conduction system thereby narrowing the QRS complex and restoring synchronous ventricular contraction. Comparison of RV pacing, CRT and CSP for various patient groups are warranted and ongoing (NCT05815745, NCT05650658). Likely, CRT as biventricular pacing will remain an important tool, also combined with CSP, should this pacing mode prove non-inferior or superior to CRT in selected patients.^36^ Importantly, our study demonstrates a prominent effect size of contemporary CRT on a wide range of endpoints in both patients with RV paced and intrinsic LBBB QRS morphology at baseline who were well-selected and well-treated with GDMT, and this serves as an important reference point for future comparisons with other pacing modalities.

### Limitations

Although prospective, this study is subject to limitations pertaining to its observational design, including risk of confounding and inability to demonstrate causality. The results apply only to patients fulfilling the RCTs’ eligibility criteria and may not translate to other CRT populations. Minor differences in eligibility criteria between the RCTs, most notably in the definition of upgrade procedures, could result in some heterogeneity. Procedural risks and lead-related complications were not investigated, as the sample size was insufficient for robust analysis. Such data have been reported in the original publications of two of the three RCTs.^13,14^ Subgroup analysis on pre-existing device type and duration of RV pacing also comprised a limited number of patients and should be interpreted cautiously. Still, this prospective study comprises a large cohort of patients from the modern CRT era with high-quality data from RCTs on a wide range of outcomes and the effect size was prominent in both groups.

## Conclusion

Patients with RV paced QRS morphology who are upgraded to a CRT device derive substantial improvement in cardiac function and functional capacity, comparable to patients with intrinsic conduction undergoing CRT.

## Supplementary Material

xvag183_Supplementary_Data

## Declarations

### Data Availability

No data were generated or analysed for this manuscript.

### Pre-registered Clinical Trial Number

None supplied.
