# Supplementary material for: Cardiac remodelling and functional status after cardiac resynchronization therapy: comparison between de-novo implantation and upgrade from right ventricular pacing
Source: ESC Heart Fail. 2026 Jun 26;13(4):xvag183. doi: 10.1093/eschf/xvag183 (PMC13344856; doi:10.1093/eschf/xvag183)
Supplement: xvag183_Supplementary_Data [file xvag183_supplementary_data.zip › Table S1.docx]

|  | Regression coefficient (%) | 95% CI | | p-value |
| --- | --- | --- | --- | --- |
| Baseline QRS morphology |  |  |  |  |
| Intrinsic conduction | ref. | ref. | | ref. |
| RV pacing | -1.5 | (-8.2 | 5.1) | 0.650 |
| Baseline QRS duration, per ms | -0.2 | (-0.3 | -0.1) | 0.002 |
| Age, per year | 0.2 | (-0.0. | 0.5) | 0.097 |
| Sex |  |  |  |  |
| Female | ref. | ref. | | ref. |
| Male | 4.1 | (-1.0 | 9.3) | 0.121 |
| Ischemic heart disease |  |  |  |  |
| No | ref. | ref. | | ref. |
| Yes | 8.4 | (3.7 | 13.0) | <0.001 |
| Atrial fibrillation |  |  |  |  |
| None | ref. | ref. | | ref. |
| Persistent or permanent | 5.6 | (-0.7 | 12.0) | 0.082 |
| Paroxysmal | 4.2 | (-1.3 | 9.6) | 0.133 |
| Trial |  |  |  |  |
| Imaging-CRT | ref. | ref. | | ref. |
| electro-crt | 11.1 | (3.9 | 18.3) | 0.003 |
| danish crt | 0.4 | (-3.9 | 4.7) | 0.866 |

**Table S1.** Multivariable linear regression analysis of covariates associated with relative change in left ventricular end-systolic volume (LVESV) from baseline to 6-month follow-up. Regression coefficients represent the adjusted mean difference in relative change in LVESV associated with each covariate while holding all other variables in the model constant. Positive coefficients indicate a smaller relative reduction in LVESV, whereas negative coefficients indicate a greater relative reduction in LVESV. For categorical variables, coefficients are interpreted relative to the indicated reference category. For continuous variables, coefficients represent the adjusted change in relative LVESV change per unit increase in the covariate. P-values test the null hypothesis that the regression coefficient equals 0.
