# Supplementary material for: Cardiac remodelling and functional status after cardiac resynchronization therapy: comparison between de-novo implantation and upgrade from right ventricular pacing
Source: ESC Heart Fail. 2026 Jun 26;13(4):xvag183. doi: 10.1093/eschf/xvag183 (PMC13344856; doi:10.1093/eschf/xvag183)
Supplement: xvag183_Supplementary_Data [file xvag183_supplementary_data.zip › Table S2.docx]

|  | **RV paced**  **(n=97)** | | **Intrinsic LBBB**  **(n=475)** | | **Intrinsic non-LBBB**  **(n=19)** | | **p-value ANOVA** | **RV paced vs LBBB, mean difference (95%Ci), p-value** | | | **RV paced vs non-LBBB, mean difference (95%Ci), p-value** | | | | **LBBB vs non-LBBB, mean difference (95%Ci), p-value** | | |
| --- | --- | --- | --- | --- | --- | --- | --- | --- | --- | --- | --- | --- | --- | --- | --- | --- | --- |
| **Biventricular pacing, %** |  |  |  |  |  |  |  |  |  |  |  |  |  |  | |  |  |
| Follow-up | 99 | (97-99) | 99 | (96-99) | 98 | (93-99) | 0.128 |  | - |  |  | - |  |  | | - |  |
| **QRS duration, ms** |  |  |  |  |  |  |  |  |  |  |  |  |  |  | |  |  |
| Baseline | 185 | ±21 | 166 | ±18 | 159 | ±15 | <0.001 | 18 | (13;23) | <0.001 | 26 | (14;37) | <0.001 | 7 | | (-3;18) | 0.295 |
| Follow-up | 146 | ±22 | 140 | ±20 | 150 | ±22 | 0.003 | 7 | (1;12) | 0.012 | -3 | (-15;9) | 1.000 | -10 | | (-21;2) | 0.121 |
| Absolute change | -38 | ±22 | -27 | ±24 | -9 | ±23 | <0.001 | -11 | (-17;-4) | <0.001 | -29 | (-44;-14) | <0.001 | -18 | | (-31;-4) | 0.007 |
| **LVEF, %** |  |  |  |  |  |  |  |  |  |  |  |  |  |  | |  |  |
| Baseline | 29 | ±6 | 27 | ±6 | 27 | ±5 | 0.014 | 2 | (0;4) | 0.012 | 2 | (-1;6) | 0.433 | 0 | | (-4;3) | 1.000 |
| Follow-up | 43 | ±9 | 40 | ±10 | 33 | ±10 | 0.001 | 3 | (0;5) | 0.040 | 9 | (3;15) | 0.001 | 7 | | (1;12) | 0.016 |
| Absolute change | 14 | ±9 | 13 | ±9 | 7 | ±9 | 0.012 | 1 | (-2;3) | 1.000 | 7 | (1;13) | 0.010 | 6 | | (1;12) | 0.014 |
| **LVESV, ml** |  |  |  |  |  |  |  |  |  |  |  |  |  |  | |  |  |
| Baseline | 131 | ±52 | 157 | ±69 | 149 | ±51 | 0.002 | -26 | (-43;-8) | 0.001 | -18 | (-58;22) | 0.825 | 7 | | (-30;45) | 1.000 |
| Follow-up | 82 | ±37 | 103 | ±58 | 128 | ±53 | <0.001 | -20 | (-35;-6) | 0.003 | -45 | (-79;-13) | 0.003 | -25 | | (-56;6) | 0.149 |
| Absolute change | -48 | ±43 | -54 | ±51 | -21 | ±34 | 0.011 | 6 | (-7;19) | 0.829 | -27 | (-57;2) | 0.080 | -33 | | (-61;-6) | 0.012 |
| Relative change (%) | -34 | ±25 | -33 | ±51 | -13 | ±24 | 0.003 | -1 | (-8;6) | 1.000 | -21 | (-37;-6) | 0.003 | -20 | | (-35;-6) | 0.003 |
| **LVEDV, ml** |  |  |  |  |  |  |  |  |  |  |  |  |  |  | |  |  |
| Baseline | 182 | ±63 | 212 | ±83 | 201 | ±58 | 0.003 | -30 | (-51;-9) | 0.002 | -19 | (-66;29) | 1.000 | 11 | | (-33;56) | 1.000 |
| Follow-up | 141 | ±50 | 164 | ±70 | 187 | ±58 | 0.002 | -23 | (-41;-5) | 0.005 | -46 | (-86;-6) | 0.018 | -23 | | (-60;15) | 0.433 |
| Absolute change | -41 | ±50 | -48 | ±70 | -14 | ±34 | 0.029 | 7 | (-9;22) | 0.864 | -27 | (-62;7) | 0.182 | -34 | | (-67;-2) | 0.036 |
| Relative change (%) | -20 | ±23 | -20 | ±25 | -5 | ±18 | 0.043 | 0 | (-7;6) | 1.000 | -14 | (-29;0) | 0.057 | -14 | | (-28;-1) | 0.038 |
| **LV mass index*, g/m^2^** |  |  |  |  |  |  |  |  |  |  |  |  |  |  | |  |  |
| Baseline | 121 | ±25 | 125 | ±34 | 136 | ±33 | 0.232 | -4 | (-16;8) | 1.000 | -16 | (-38;6) | 0.264 | -12 | | (-32;9) | 0.491 |
| Follow-up | 108 | ±27 | 109 | ±33 | 139 | ±35 | 0.001 | -1 | (-13;17) | 1.000 | -31 | (-53;-9) | 0.002 | -30 | | (-49;-10) | 0.001 |
| Absolute change | -13 | ±25 | -16 | ±30 | 2 | ±32 | 0.056 | 3 | (-7;14) | 1.000 | -15 | (-35;5) | 0.224 | -18 | | (-37;0) | 0.054 |
| **LA volume index*, ml/m^2^** |  |  |  |  |  |  |  |  |  |  |  |  |  |  | |  |  |
| Baseline | 42 | ±19 | 37 | ±17 | 36 | ±13 | 0.169 | 5 | (-2;12) | 0.207 | 6 | (-6;18) | 0.634 | 1 | | (-10;12) | 1.000 |
| Follow-up | 43 | ±21 | 36 | ±16 | 37 | ±12 | 0.031 | 7 | (1;14) | 0.025 | 6 | (-6;19) | 0.711 | -1 | | (-13;10) | 1.000 |
| Absolute change | 1 | ±12 | -1 | ±11 | 1 | ±10 | 0.397 | 2 | (-2;7) | 0.559 | 1 | (-7;9) | 1.000 | -1 | | (-9;-6) | 1.000 |
| **NYHA functional class** |  |  |  |  |  |  |  |  |  |  |  |  |  |  | |  |  |
| Baseline I/II/III or IVa, n(%) | 0(0)/42(43)/55(57) | | 0(0)/248(52)/227(48) | | 0(0)/7(37)/12(63) | | 0.212 | - |  |  | - |  |  | - | |  |  |
| Follow-up I/II/III or IVa, n(%) | 25(26)/53(56)/17(18) | | 158(33)/259(53)  /57(12) | | 7(37)/10(52)/2(11) | | 0.543 | - |  |  | - |  |  | - | |  |  |
| ≥1 improvement, n(%) | 57 | (60) | 304 | (64) | 15 | (79) | 0.240 | - |  |  | - |  |  | - | |  |  |
| **Quality of Life** |  |  |  |  |  |  |  |  |  |  |  |  |  |  | |  |  |
| *KCCQ12* |  |  |  |  |  |  |  |  |  |  |  |  |  |  | |  |  |
| Baseline | 54 | ±24 | 58 | ±19 | 52 | ±23 | 0.065 | - |  |  | - |  |  | - | |  |  |
| Follow-up | 68 | ±20 | 73 | ±18 | 62 | ±23 | 0.223 | - |  |  | - |  |  | - | |  |  |
| Absolute change | 16 | ±22 | 14 | ±18 | 9 | ±21 | 0.070 | - |  |  | - |  |  | - | |  |  |
| **Loop diuretics, mg** |  |  |  |  |  |  |  |  |  |  |  |  |  |  | |  |  |
| Baseline | 60 | (40-120) | 60 | (40-80) | 80 | (80-120) | 0.012 | - |  |  | - |  |  | - | |  |  |
| Follow-up | 40 | (40-120) | 40 | (40-80) | 80 | (40-120) | 0.292 | - |  |  | - |  |  | - | |  |  |
| Dose reduction, n(%) | 23 | (33) | 92 | (27) | 9 | (53) | 0.062 | - |  |  | - |  |  | - | |  |  |

**Table S2.** Endpoints at baseline and 6 months follow-up and change from baseline to 6 months follow-up reported by QRS morphology. One-way ANOVA was used to test overall between-group differences in case of normal distribution and Kruskall-Wallis test in absence of normal distribution. Pairwise comparisons are reported as mean differences with Bonferroni corrected 95% confidence intervals and test using independent t-test with Bonferroni correction. If there was no significant difference in the primary analysis, we did not proceed with pairwise comparison. One patient with intrinsic conduction had missing data on QRS morphology. Minnesota Living with Heart Failure questionnaire, 6-minute-walk-test and NT-proBNP were not available for non-LBBB patients.
