# Supplementary material for: Cardiac remodelling and functional status after cardiac resynchronization therapy: comparison between de-novo implantation and upgrade from right ventricular pacing
Source: ESC Heart Fail. 2026 Jun 26;13(4):xvag183. doi: 10.1093/eschf/xvag183 (PMC13344856; doi:10.1093/eschf/xvag183)
Supplement: xvag183_Supplementary_Data [file xvag183_supplementary_data.zip › Table S3.docx]

|  | **RV paced**  **(n=92)** | | **Intrinsic**  **(n=470)** | | **Difference**  **(95% CI)** | | **p-value** |
| --- | --- | --- | --- | --- | --- | --- | --- |
| **Biventricular pacing, %** |  |  |  |  |  |  |  |
| Follow-up | 99 | (97-100) | 99 | (96-99) |  | - | 0.066 |
| **QRS duration, ms** |  |  |  |  |  |  |  |
| Baseline | 184 | ±21 | 166 | ±18 | 18 | (14;22) | <0.001 |
| Follow-up | 146 | ±22 | 140 | ±20 | 6 | (1;10) | 0.018 |
| Absolute change | -37 | ±27 | -26 | ±24 | -12 | (-17;-6) | <0.001 |
| **LVEF, %** |  |  |  |  |  |  |  |
| Baseline | 28 | ±5 | 26 | ±6 | 2 | ( 1;3) | <0.001 |
| Follow-up | 43 | ±9 | 39 | ±10 | 3 | ( 1;6) | 0.004 |
| Absolute change | 14 | ±9 | 13 | ±10 | 1 | (-1;3) | 0.280 |
| **LVESV, ml** |  |  |  |  |  |  |  |
| Baseline | 134 | ±51 | 160 | ±68 | -26 | (-40;-11) | <0.001 |
| Follow-up | 84 | ±37 | 106 | ±58 | -21 | (-34;-9) | <0.001 |
| Absolute change | -50 | ±43 | -55 | ±51 | 5 | (-6;15) | 0.396 |
| Relative change (%) | -35 | ±25 | -33 | ±25 | -2 | (-7;4) | 0.559 |
| **LVEDV, ml** |  |  |  |  |  |  |  |
| Baseline | 186 | ±62 | 214 | ±82 | -28 | (-46;-10) | 0.003 |
| Follow-up | 144 | ±49 | 167 | ±70 | -23 | (-38;-7) | 0.003 |
| Absolute change | -42 | ±51 | -48 | ±60 | 6 | (-7;19) | 0.363 |
| Relative change (%) | -20 | ±23 | -20 | ±24 | 0 | (-5;5) | 0.967 |
| **LV mass index*, g/m^2^** |  |  |  |  |  |  |  |
| Baseline | 121 | ±25 | 126 | ±34 | -5 | (-15;5) | 0.322 |
| Follow-up | 108 | ±26 | 111 | ±34 | -3 | (-13;7) | 0.525 |
| Absolute change | -13 | ±25 | -14 | ±31 | 2 | (-7;10) | 0.697 |
| **LA volume index*, ml/m^2^** |  |  |  |  |  |  |  |
| Baseline | 43 | ±18 | 37 | ±17 | 6 | (0;11) | 0.046 |
| Follow-up | 44 | ±21 | 36 | ±16 | 7 | (2;13) | 0.005 |
| Absolute change | 2 | ±12 | -1 | ±11 | 2 | (-1;6) | 0.186 |
| **NT-proBNP**, ng/l** |  |  |  |  |  |  |  |
| Baseline | 2,011 | (1,035-3,319) | 1,392 | (638-3,164) | 0.66 | (0.43;1.01) | 0.056 |
| Follow-up | 1,059 | (762-1,958) | 685 | (263-1,640) | 0.60 | (0.37;0.97) | 0.038 |
| Relative change (GMR) | 0.51 | (0.39-0.66) | 0. 48 | (0.42-0.54) | 0.93 | (0.66;1.32) | 0.703 |
| **6MWT*, m** |  |  |  |  |  |  |  |
| Baseline | 345 | ±149 | 395 | ±109 | -50 | (-93;-6) | 0.029 |
| Follow-up | 405 | ±103 | 438 | ±99 | -34 | (-59;-9) | 0.009 |
| Absolute change | 63 | ±67 | 43 | ±66 | 19 | (-6;46) | 0.137 |
| **NYHA functional class** |  |  |  |  |  |  |  |
| Baseline I/II/III or IVa, n(%) | 0(0)/42(46)/50(54) | | 0(0)/241(51)/219(49) | |  | - | 0.481 |
| Follow-up I/II/III or IVa, n(%) | 25(28)/49(54)/16(18) | | 155(33)/257(55)/57(12) | |  | - | 0.381 |
| ≥1 improvement, n(%) | 53 | (58) | 304 | (65) |  | - | 0.236 |
| **Quality of Life** |  |  |  |  |  |  |  |
| *MLWHF* |  |  |  |  |  |  |  |
| Baseline | 36 | ±21 | 35 | ±22 | 1 | (-7;10) | 0.725 |
| Follow-up | 21 | ±19 | 20 | ±19 | 0 | (-7;8) | 0.878 |
| Absolute change | -16 | ±26 | -14 | ±18 | -2 | (-9;5) | 0.595 |
| *KCCQ12* |  |  |  |  |  |  |  |
| Baseline | 54 | ±24 | 58 | ±19 | -4 | (-10;2) | 0.237 |
| Follow-up | 68 | ±21 | 72 | ±19 | -4 | (-10;1) | 0.144 |
| Absolute change | 15 | ±23 | 14 | ±18 | 1 | (-4;7) | 0.641 |
| *Standardized change, Δ z-score* | 0.74 | ±1.13 | 0.66 | ±0.86 | 0.09 | (-0.12;0.30) | 0.411 |
| **Loop diuretics, mg** |  |  |  |  |  |  |  |
| Baseline | 60 | (40-100) | 80 | (40-120) |  | - | 0.743 |
| Follow-up | 40 | (40-120) | 40 | (40-80) |  | - | 0.787 |
| Dose reduction, n(%) | 23 | (35) | 97 | (29) |  | - | 0.303 |
|  | | | | | | | |

**Table S3.** Endpoints at baseline and 6 months follow-up and change from baseline to 6 months follow-up reported for patients with RV pacing and intrinsic conduction, all with LVEF≤35% at baseline.
