# Supplementary material for: Cardiac remodelling and functional status after cardiac resynchronization therapy: comparison between de-novo implantation and upgrade from right ventricular pacing
Source: ESC Heart Fail. 2026 Jun 26;13(4):xvag183. doi: 10.1093/eschf/xvag183 (PMC13344856; doi:10.1093/eschf/xvag183)
Supplement: xvag183_Supplementary_Data [file xvag183_supplementary_data.zip › Table S4.docx]

|  | **Pacemaker**  **(n=93)** | | **ICD**  **(n=68)** | | **Difference**  **(95% CI)** | | **p-value** |
| --- | --- | --- | --- | --- | --- | --- | --- |
| **Biventricular pacing, %** |  |  |  |  |  |  |  |
| Follow-up | 99 | (97-99) | 98 | (93-99) |  | - | 0.002 |
| **QRS duration, ms** |  |  |  |  |  |  |  |
| Baseline | 181 | ±20 | 172 | ±23 | 9 | (3;16) | 0.007 |
| Follow-up | 142 | ±18 | 150 | ±22 | -8 | (-14;-2) | 0.011 |
| Absolute change | -39 | ±24 | -21 | ±28 | -18 | (-26;-9) | <0.001 |
| **LVESV, ml** |  |  |  |  |  |  |  |
| Baseline | 131 | ±54 | 161 | ±73 | -29 | (-49;-10) | 0.004 |
| Follow-up | 80 | ±36 | 130 | ±66 | -50 | (-66;-34) | <0.001 |
| Absolute change | -52 | ±41 | -31 | ±44 | -21 | (-34;-7) | 0.003 |
| Relative change (%) | -37 | ±21 | -17 | ±28 | -20 | (-28;-12) | <0.001 |
| **LVEDV, ml** |  |  |  |  |  |  |  |
| Baseline | 182 | ±64 | 217 | ±86 | -34 | (-58;-11) | 0.004 |
| Follow-up | 136 | ±48 | 196 | ±77 | -60 | (-80;-41) | <0.001 |
| Absolute change | -47 | ±49 | -21 | ±55 | -26 | (-42;-9) | 0.002 |
| Relative change (%) | -23 | ±20 | -6 | ±27 | -17 | (-24;-9) | <0.001 |
| **LVEF, %** |  |  |  |  |  |  |  |
| Baseline | 29 | ±6 | 27 | ±6 | 2 | ( 0;4) | 0.017 |
| Follow-up | 42 | ±9 | 36 | ±9 | 7 | ( 4;10) | <0.001 |
| Absolute change | 13 | ±9 | 9 | ±8 | 5 | (2;7) | 0.001 |
| **LV mass index*, g/m^2^** |  |  |  |  |  |  |  |
| Baseline | 122 | ±26 | 127 | ±37 | -4 | (-17;9) | 0.570 |
| Follow-up | 108 | ±26 | 123 | ±31 | -15 | (-27;-3) | 0.015 |
| Absolute change | -14 | ±25 | -2 | ±22 | -11 | (-22;-1) | 0.030 |
| **LA volume index*, ml/m^2^** |  |  |  |  |  |  |  |
| Baseline | 39 | ±14 | 45 | ±20 | -6 | (-13;2) | 0.137 |
| Follow-up | 39 | ±13 | 47 | ±25 | -8 | (-17;1) | 0.052 |
| Absolute change | 1 | ±9 | 2 | ±12 | -1 | (-6;4) | 0.624 |
| **NT-proBNP**, ng/l** |  |  |  |  |  |  |  |
| Baseline | 1,884 | (1,495-2,374) | 1,585 | (1,226-2,048) | 1.19 | (0.84;1.68) | 0.321 |
| Follow-up | 1,045 | (761-1,436) | 1,209 | (753-1,942) | 0.86 | (0.51;1.48) | 0.590 |
| Relative change (GMR) | 0.46 | (0.34;0.62) | 0.75 | (0.58;0.97) | 0.61 | (0.41;0.92) | 0.018 |
| **6MWT*, m** |  |  |  |  |  |  |  |
| Baseline | 354 | ±129 | 369 | ±149 | -15 | (-91;62) | 0.707 |
| Follow-up | 402 | ±102 | 420 | ±92 | -18 | (-53;17) | 0.319 |
| Absolute change | 57 | ±62 | 56 | ±20 | 1 | (-44;45) | 0.982 |
| **NYHA functional class** |  |  |  |  |  |  |  |
| Baseline I/II/III or IVa, n(%) | 0(0)/37(40)/56(60) | | 0(0)/34(50)/34(50) | |  | - | 0.053 |
| Follow-up I/II/III or IVa, n(%) | 26(29)/47(52)/18(20) | | 15(22)/43(63)/10(15) | |  | - | 0.245 |
| ≥1 improvement, n(%) | 54 | (59) | 40 | (59) |  | - | 0.538 |
| **Quality of Life** |  |  |  |  |  |  |  |
| *MLWHF* |  |  |  |  |  |  |  |
| Baseline | 35 | ±21 | 35 | ±23 | 0 | (-12;12) | 0.956 |
| Follow-up | 22 | ±20 | 17 | ±18 | 5 | (-6;15) | 0.367 |
| Absolute change | -13 | ±23 | -18 | ±20 | 5 | (-7:17) | 0.412 |
| *KCCQ12* |  |  |  |  |  |  |  |
| Baseline | 54 | ±22 | 58 | ±23 | -4 | (-13;5) | 0.404 |
| Follow-up | 67 | ±21 | 68 | ±18 | -2 | (-9;6) | 0.658 |
| Absolute change | 14 | ±23 | 10 | ±17 | 4 | (-4;13) | 0.390 |
| *Standardized change, Δ z-score* | 0.62 | ±1.10 | 0.59 | ±0.85 | 0.02 | (-0.30;0.35) | 0.879 |
| **Loop diuretics, mg** |  |  |  |  |  |  |  |
| Baseline | 40 | (40-80) | 80 | (40-120) |  | - |  |
| Follow-up | 40 | (30-80) | 40 | (40-100) |  | - |  |
| Dose reduction, n(%) | 23 | (33) | 13 | (27) |  | - | 0.547 |

**Table S4.** Endpoints at baseline and follow-up and change from baseline to follow-up for patients with pre-existing pacemaker and ICD, irrespective of RV pacing or intrinsic conduction at baseline, and between-group difference with 95% confidence intervals. Data are presented as mean (SD) or median (IQR) for continuous variables pending normal distribution and n (%) for categorical measures. Between-group differences are assessed using unpaired t-test in case of normal distribution, the Wilcoxon rank-sum test in absence of normal distribution and Pearson’s chi-square test for categorical variables. Within-group differences are assessed using the paired t-test in case of normal distribution and the Wilcoxon signed rank test in absence of normal distribution. *Data not available from all trials. **The median ratio was calculated as the geometrical mean ratio = the mean difference on the log scale. The median ratio may differ from the ratio of raw medians due to distributional asymmetry. QoL was assessed with MLWHF (range 0-105, lower scores reflect better QoL) and KCCQ12 (range 0-100, higher values indicate better QoL) and standardized Δ z-score using the baseline standard deviation (positive values indicate improvement) was used to enable comparison across instruments. KCCQ12 Kansas City Cardiomyopathy Questionaire 12, LVEDV left ventricular end-diastolic volume, LVEF left ventricular ejection fraction, LVESV left ventricular end-systolic volume, NT-proBNP N-terminal pro-Brain Natriuretic Peptide, NYHA New York Heart Association, 6MWT Six-minute walk test, MLWHF Minnesota Living with Heart Failure.
