# Supplementary material for: Cardiac remodelling and functional status after cardiac resynchronization therapy: comparison between de-novo implantation and upgrade from right ventricular pacing
Source: ESC Heart Fail. 2026 Jun 26;13(4):xvag183. doi: 10.1093/eschf/xvag183 (PMC13344856; doi:10.1093/eschf/xvag183)
Supplement: xvag183_Supplementary_Data [file xvag183_supplementary_data.zip › Table S5.docx]

|  | **Earlier than 4.5 years**  **(n=48)** | | **Later than 4.5 years**  **(n=49)** | | **Difference**  **(95% CI)** | | **p-value** |
| --- | --- | --- | --- | --- | --- | --- | --- |
| **Biventricular pacing, %** |  |  |  |  |  |  |  |
| Follow-up | 99 | (97-99) | 99 | (97-100) |  | - | 0.699 |
| **QRS duration, ms** |  |  |  |  |  |  |  |
| Baseline | 183 | ±20 | 186 | ±19 | -4 | (-12;4) | 0.357 |
| Follow-up | 149 | ±23 | 144 | ±20 | 4 | (-4;13) | 0.011 |
| Absolute change | -33 | ±27 | -42 | ±26 | 8 | (-2;19) | 0.126 |
| **LVESV, ml** |  |  |  |  |  |  |  |
| Baseline | 118 | ±42 | 144 | ±58 | -26 | (-46;-5) | 0.014 |
| Follow-up | 79 | ±34 | 86 | ±40 | -7 | (-22;7) | 0.330 |
| Absolute change | -39 | ±37 | -58 | ±46 | 19 | (2;36) | 0.032 |
| Relative change (%) | -32 | ±25 | -36 | ±25 | 5 | (-5;14) | 0.372 |
| **LVEDV, ml** |  |  |  |  |  |  |  |
| Baseline | 169 | ±52 | 195 | ±70 | -26 | (-52;-1) | 0.038 |
| Follow-up | 138 | ±49 | 144 | ±52 | -6 | (-25;15) | 0.589 |
| Absolute change | -31 | ±42 | -51 | ±55 | 21 | (1;41) | 0.041 |
| Relative change (%) | -17 | ±23 | -23 | ±23 | 6 | (-3;15) | 0.227 |
| **LVEF, %** |  |  |  |  |  |  |  |
| Baseline | 31 | ±5 | 27 | ±6 | 4 | (2;6) | 0.001 |
| Follow-up | 44 | ±9 | 41 | ±9 | 3 | (-1;6) | 0.126 |
| Absolute change | 13 | ±9 | 14 | ±9 | 1 | (-5;3) | 0.606 |
| **LV mass index*, g/m^2^** |  |  |  |  |  |  |  |
| Baseline | 121 | ±25 | 120 | ±25 | 2 | (-12;16) | 0.803 |
| Follow-up | 114 | ±24 | 102 | ±28 | 12 | (-3;26) | 0.104 |
| Absolute change | -7 | ±19 | -17 | ±28 | 10 | (-3;24) | 0.138 |
| **LA volume index*, ml/m^2^** |  |  |  |  |  |  |  |
| Baseline | 39 | ±15 | 46 | ±21 | -7 | (-18;4) | 0.186 |
| Follow-up | 40 | ±13 | 47 | ±26 | -7 | (-19;5) | 0.231 |
| Absolute change | 1 | ±8 | 2 | ±12 | 0 | (-7;7) | 0.975 |
| **NT-proBNP**, ng/l** |  |  |  |  |  |  |  |
| Baseline | 1,610 | (670-2,515) | 2,320 | (1,169-4,261) | 0.57 | (0.36;0.89) | 0.014 |
| Follow-up | 1,055 | (647-1,510) | 1,515 | (867-2,713) | 0.69 | (0.39;1.24) | 0.206 |
| Relative change (GMR) | 0.57 | (0.41;0.79) | 0.55 | (0.37;0.83) | 1.02 | (0.60;1.73) | 0.930 |
| **6MWT*, m** |  |  |  |  |  |  |  |
| Baseline | 321 | ±139 | 353 | ±148 | -32 | (-135;73) | 0.541 |
| Follow-up | 372 | ±116 | 417 | ±134 | -44 | (-136;47) | 0.327 |
| Absolute change | 52 | ±66 | 64 | ±66 | -13 | (-60;35) | 0.587 |
| **NYHA functional class** |  |  |  |  |  |  |  |
| Baseline I/II/III or IVa, n(%) | 0(0)/21(44)/57(56) | | 0(0)/21(43)/28(57) | |  | - | 1.000 |
| Follow-up I/II/III or IVa, n(%) | 9(19)/29(62)/9(19) | | 16(33)/24(50)/8(17) | |  | - | 0.284 |
| ≥1 improvement, n(%) | 25 | (53) | 32 | (67) |  | - | 0.212 |
| **Quality of Life** |  |  |  |  |  |  |  |
| *MLWHF* |  |  |  |  |  |  |  |
| Baseline | 35 | ±21 | 35 | ±23 | 0 | (-12;12) | 0.956 |
| Follow-up | 22 | ±20 | 17 | ±18 | 5 | (-6;15) | 0.367 |
| Absolute change | -13 | ±23 | -18 | ±20 | 5 | (-7:17) | 0.412 |
| *KCCQ12* |  |  |  |  |  |  |  |
| Baseline | 54 | ±22 | 58 | ±23 | -4 | (-13;5) | 0.404 |
| Follow-up | 67 | ±21 | 68 | ±18 | -2 | (-9;6) | 0.658 |
| Absolute change | 14 | ±23 | 10 | ±17 | 4 | (-4;13) | 0.390 |
| *Standardized change, Δ z-score* | 0.61 | ±1.01 | 0.76 | ±1.08 | -0.15 | (-0.59;0.29) | 0.491 |
| **Loop diuretics, mg** |  |  |  |  |  |  |  |
| Baseline | 60 | (40-120) | 60 | (40-80) |  | - |  |
| Follow-up | 40 | (40-120) | 40 | (20-80) |  | - |  |
| Dose reduction, n(%) | 5 | (16) | 18 | (48) |  | - | 0.005 |

**Table S5.** Endpoints at baseline and follow-up and change from baseline to follow-up for patients with duration of RV pacing shorter and longer than the median duration from the patient’s first device to CRT upgrade (4.5 years) and between-group difference with 95% confidence intervals. Data are presented as mean (SD) or median (IQR) for continuous variables pending normal distribution and n (%) for categorical measures. Between-group differences are assessed using unpaired t-test in case of normal distribution, the Wilcoxon rank-sum test in absence of normal distribution and Pearson’s chi-square test for categorical variables. Within-group differences are assessed using the paired t-test in case of normal distribution and the Wilcoxon signed rank test in absence of normal distribution. *Data not available from all trials. **The median ratio was calculated as the geometrical mean ratio = the mean difference on the log scale. The median ratio may differ from the ratio of raw medians due to distributional asymmetry. QoL was assessed with MLWHF (range 0-105, lower scores reflect better QoL) and KCCQ12 (range 0-100, higher values indicate better QoL) and standardized Δ z-score using the baseline standard deviation (positive values indicate improvement) was used to enable comparison across instruments. KCCQ12 Kansas City Cardiomyopathy Questionaire 12, LVEDV left ventricular end-diastolic volume, LVEF left ventricular ejection fraction, LVESV left ventricular end-systolic volume, NT-proBNP N-terminal pro-Brain Natriuretic Peptide, NYHA New York Heart Association, 6MWT Six-minute walk test, MLWHF Minnesota Living with Heart Failure.
